# MicroRNA Signatures of Drought Signaling in Rice Root

**DOI:** 10.1371/journal.pone.0156814

**Published:** 2016-06-08

**Authors:** Behnam Bakhshi, Ehsan Mohseni Fard, Nava Nikpay, Mohammad Ali Ebrahimi, Mohammad Reza Bihamta, Mohsen Mardi, Ghasem Hosseini Salekdeh

**Affiliations:** 1 Department of Systems Biology, Agricultural Biotechnology Research Institute of Iran, Agricultural Research, Education, and Extension Organization, Karaj, Iran; 2 Department of Plant Breeding, Science and Research Branch, Islamic Azad University, Tehran, Iran; 3 Department of Agronomy and Plant Breeding, Faculty of Agriculture, University of Zanjan, Zanjan, Iran; 4 Department of Biotechnology, Payame Noor University, Tehran, Iran; 5 Department of Agronomy and Plant Breeding, Faculty of Agricultural Science and Engineering, College of Agriculture and Natural Resources, University of Tehran, Karaj, Iran; 6 Department of Molecular Systems Biology, Cell Science Research Center, Royan Institute for Stem Cell Biology and Technology, ACECR, Tehran, Iran; Universidade Federal do Rio Grande do Sul, BRAZIL

## Abstract

**Background:**

Drought stress is one of the most important abiotic stresses and the main constraint to rice agriculture. MicroRNA-mediated post-transcriptional gene regulation is one of the ways to establish drought stress tolerance in plants. MiRNAs are 20–24-nt regulatory RNAs that play an important role in regulating plant gene expression upon exposure to biotic and abiotic stresses.

**Methodology/Principal Findings:**

In this study, we applied a partial root drying system as well as a complete root drying system to identify miRNAs involved in conditions of drought stress, drought signaling and wet signaling using high-throughput sequencing. To this end, we produced four small RNA libraries: (1) fully-watered (WW), (2) fully-droughted (WD), and split-root systems where (3) one-half was well watered (SpWW) and (4) the other half was water-deprived (SpWD). Our analysis revealed 10,671 and 783 unique known and novel miRNA reads in all libraries, respectively. We identified, 65 (52 known + 13 novel), 72 (61 known + 11 novel) and 51 (38 known + 13 novel) miRNAs that showed differential expression under conditions of drought stress, drought signaling and wet signaling, respectively. The results of quantitative real-time PCR showed expression patterns similar to the high-throughput sequencing results. Furthermore, our target prediction led to the identification of 244, 341 and 239 unique target genes for drought-stress-, drought-signaling- and wet-signaling-responsive miRNAs, respectively.

**Conclusions/Significance:**

Our results suggest that miRNAs that are responsive under different conditions could play different roles in the regulation of abscisic acid signaling, calcium signaling, detoxification and lateral root formation.

## Introduction

Drought stress is one of the major abiotic stresses that plants face. Response to this stress involves complex mechanisms of signal transduction. To date the molecular mechanisms by which plant genes are regulated under drought stress is still unclear [1]. Gene expression could be regulated at the post-transcriptional level through small RNAs such as microRNAs (miRNAs) and small interfering RNAs (siRNAs) [2]. Since the first discovery of miRNAs in the form of Lin-4 and let-7 in *Caenorhabditis elegans* [3] and 16 miRNAs in *Arabidopsis* [4], miRNAs have been considered as important family of regulatory molecules in plants. These regulatory molecules usually target transcription factors and are involved in the control of several cellular processes, such as adaptation to biotic and abiotic stresses via their positive or negative regulatory roles [5,6]. Thus, miRNAs could be considered as core of regulatory expression networks. MiRNAs are transcribed by RNA polymerase II from non-protein coding transcription units as primary miRNAs(pri-miRNAs) with a secondary hairpin structures [7,8]. After two steps of pri-miRNAs processing, mature miRNAs are generated [9]. Dicer-like 1 (DCL1) from the RNAse III enzyme family play a key role in these two steps by the cleavage of pri-miRNA to precursor miRNA (pre-miRNA) in the nucleus and then the cleavage of pre-miRNA to miRNA/miRNA* in the cytoplasm [9,10]. Plant miRNAs are usually 20–24 nt in length [11]. MiRNA is loaded into the argonaut component of the RNA-induced silencing complex (RISC) in the cytoplasm, which acts as endoplasmic cutter of the mRNA target [12].

Using earlier technologies such as microarray or northern blot, researchers could study the role of miRNAs in drought stress. For example, Zhao et al. introduced five drought-responsive miRNAs in shoot and root of rice using microarray and northern blot analyses [13]. In addition, Zhou et al. used microarray to detect 30 drought-responsive miRNAs involved in tillering and reproductive stages of rice [14]. As searches for drought-responsive miRNAs in rice continued, technological advances made researchers interested in using high-throughput approaches such as Illumina technology to achieve more accurate surveys of novel miRNAs. This can be exemplified by studies in which high-throughput technologies have been applied to study the role of miRNAs in development of rice grains, leading to the identification of novel miRNAs in rice [15,16,17]. High throughput technology could also identify miRNAs involved in drought stress responses. As an example, Jian et al. detected novel miRNAs in rice using the 3730 DNA analyzer, four of which showed altered expression under drought stress [18]. Additionally, Barrera-Figueroa recently found 18 drought-responsive miRNAs in rice using high-throughput technologies [19].

Although root is the primary sensor of water status [20], but using high-throughput technologies, few studies have been done to identify drought-responsive miRNAs in roots of rice. It was reported that drought stress leads to a reduction in yield, even in parts of the root system that only had been partially dried [21]. Our previous reports show that, partial drying treatments (split-root system) leads to an increase in abscisic acid (ABA) concentration compared with well-watered condition. However, this concentration was lower than fully-droughted condition [22]. Thus, reduction in the plant yield under partial drying could be due to the existence of drought signals that are mediated by plant hormones such as ABA. In this study, we applied a partial root drying method as well as a full drying method in order to identify miRNAs involved in conditions of drought, drought signaling and wet signaling, using high-throughput deep sequencing technology. This is a continuation of our previous studies in which partial root drying was applied to identify drought-, drought-signaling- and wet-signaling-responsive proteomes in the shoot [23] and root [22] of rice. The approach of this study was to compare four root tissues: (1) fully-watered (well-watered, WW), (2) fully-droughted (water-deprived, WD), and split-root systems in which (3) one-half was well-watered (SpWW) and (4) the other half was water-deprived (SpWD). We were interested to address two concepts. Firstly, how wet roots in SpWW affected the expression of miRNAs in dried roots of SpWD hydraulically by sending wet signals. Secondly, how the expression of miRNAs in wet roots of SpWW was altered by drought signals sent from adjacent dried roots of SpWD. Thus, by comparing WW with SpWW, we could identify miRNAs associated with drought signaling. Furthermore, comparison between WD and SpWD helped us to identify miRNAs involved in wet signaling. In addition, by comparing WW with WD, we could identify miRNAs associated with drought stress.

## Materials and Methods

### Plant materials and sample preparation

**Fig 1:**
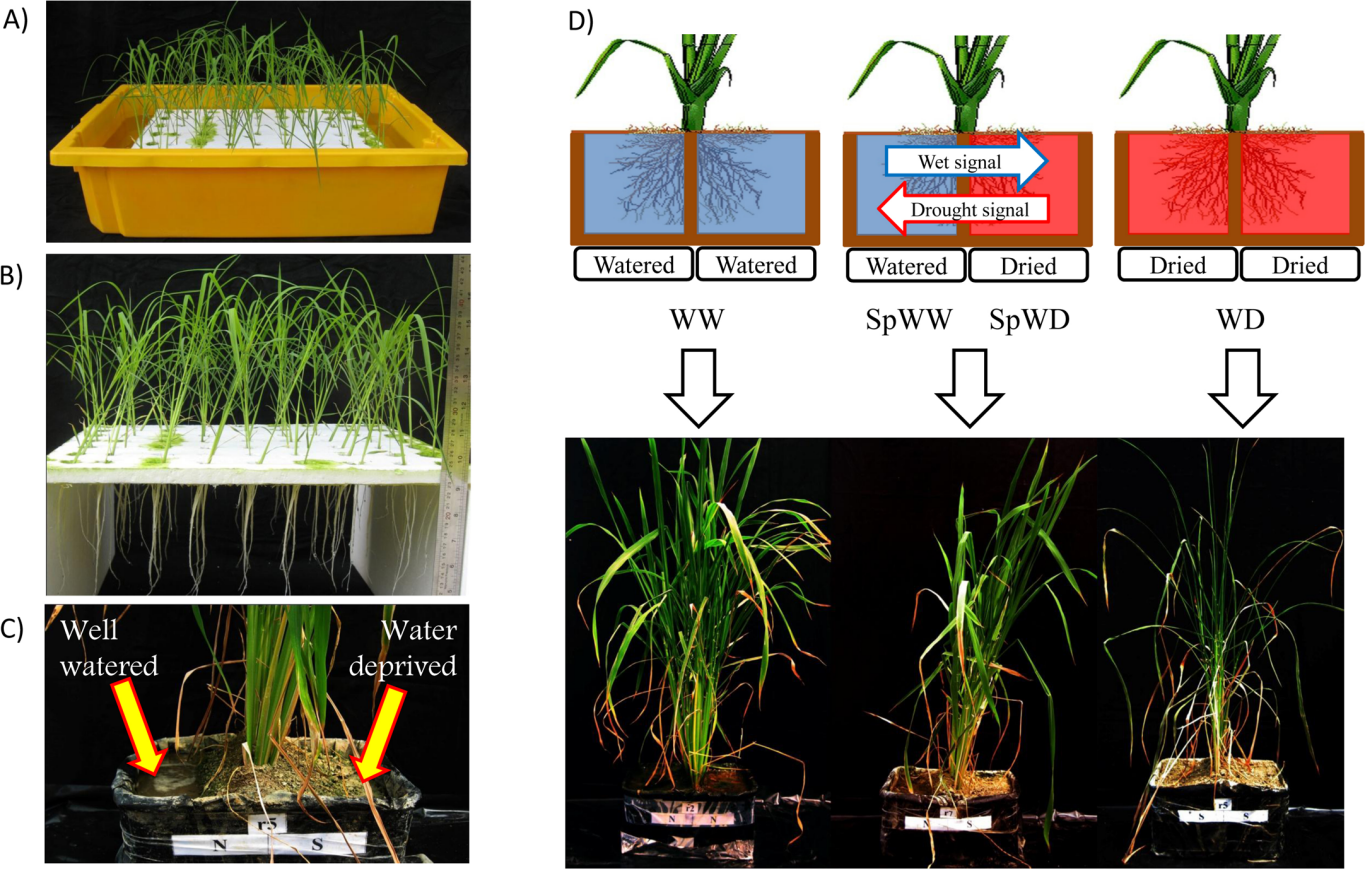
Sample preparation steps in the greenhouse. (A) Rice seedlings with Yoshida culture solution. (B) Rice roots before transplantation. (C) Split-root system of watering. (D) Three watering treatments used in this study.

Seeds of *Oryza sativa*, genotype IR64, were obtained from the International Rice Research Institute (Los Baños, The Philippines). These seeds were surface-sterilized, pre-germinated for 3 d, grown on Yoshida culture solution for 10 d (Fig 1A and 1B) and then transplanted into rectangular pots [measuring 26 cm (length) × 20 cm (width) × 15 cm (height)] for split-root culture. The pots had two equal volumes that were separated by an internal wall, which allowed us to subject these two different parts to identical or different levels of watering. A mixture of loam, sand and clay (1:2:1) was added to pots to within 5cm from the top of both parts. Seedling roots were equally distributed between the two parts of the pot. The plants were fully-watered for 14 d after transplantation. Then, the roots of seedlings were subjected to three treatments for 14 d with three replications: a) plants in which both parts were fully-watered daily (well-watered, WW); b) plants in which watering was stopped in both parts (water-deprived, WD); and c) plants in which watering was stopped only in one out of two parts with one drying root half (SpWD) and one well-watered root half (SpWW) (Fig 1C and 1D). Experiments were carried out in a greenhouse in spring-summer period, with natural illumination at Agricultural Biotechnology Research Institute. Urea (2.73 g), solophos (1.84 g) and muriate of potash (1.04 g) were added to each pot, as sources of nitrogen, phosphate and potassium, respectively. Roots of each part of the pots were sampled and washed very briefly with water. Root tissues were immediately frozen in liquid nitrogen. It was then stored at −80°C.

### Small RNA library construction for high-throughput sequencing

The RNA from 100-mg root samples under WW, WD, SpWW and SpWD conditions was isolated using a High Pure miRNA Isolation Kit (Roche, Germany) in accordance with the manufacturer’s instructions and quantified using an ND-1000 spectrophotometer (NanoDrop; Thermo Scientific, USA). The pooled miRNA of three replications from different root conditions was used to construct libraries of small RNA, and sequencing was conducted using an Illumina HiSeq2000 (Beijing Genomics Institute, China), following standard protocols.

### Data analysis

Data cleaning was performed on 50-nt sequencing tags, which included the removal of low-quality reads, reads with 5' primer contaminants, reads without a 3' primer, reads without the insert tag, reads with poly-A sequences and reads shorter than 18 nt. Subsequently, small RNAs were mapped in the genome in order to show their genomic distribution using SOAP. Then, the corresponding non-coding RNA sequences (rRNAs, tRNAs, snRNAs and snoRNAs) were excluded using GenBank (https://www.ncbi.nlm.nih.gov/genbank/) and Rfam (http://www.sangar.ac.uk/software/Rfam). All remaining small RNA sequences were aligned with registered miRNAs in miRBase version 17.0 (http://mirbase.org/) to detect known miRNAs. These standard bioinformatic analyses classified the clean tags into different categories: repeat-associated RNAs, rRNAs, tRNAs, snRNAs, snoRNAs, exons and introns, known miRNA and unannotated sRNAs. Finally, the prediction software Mireap (http://sourceforge.net/project/mireap/) was used to predict novel miRNAs by exploring the secondary structure, the dicer cleavage site and the minimum free energy of the unannotated small RNA tags that could be mapped in the genome [24].

The normalized miRNA expression level (the absolute count of miRNAs/total count of miRNAs × 1,000,000) was used to compare differential expression of miRNAs among WW, WD, SpWW and SpWD libraries. All miRNAs that showed a normalized read count of less than 10 transcript per million (TPM) in all libraries were excluded from further analysis. A miRNA was identified as being ‘responsive’ only if the following two criteria were met: (1) the miRNA expression level showed more than two fold change (log_2_ ratio was greater than 1 or less than -1); and (2) the difference in expression was significant according to the method of Audic and Claverie [25] (p-value < 0.05).

Our study focused on miRNA family members as well. For example, miR172a, miR172b, miR172c and miR172d were studied individually for their expression and target prediction instead of studying miR172 family in general to emphasis different expression patterns of each family member.

Potential target genes were predicted for miRNAs using psRNATarget (http://plantgrn.noble.org/psRNATarget/) by using the miRNA sequences as queries for a search against *Oryza sativa* TIGR genome cDNA OSA1 release 5.0. The maximum expectation value for miRNA target prediction was considered up to three times. All predicted targets were searched against starBase degradom database [26] and also previous degradom sequencing published data in rice plant [27,28]. In addition, target gene ontology analysis for the miRNAs was performed using the rice genome project database (rice.plantbiology.msu.edu).

### Validation of the miRNA expression profiles via stem-loop RT-PCR

cDNA synthesis was carried out using an RNA template and reverse transcriptase (Invitrogen, USA). Primers for stem-loop RT-PCR and gene-specific real-time PCR primers (S1 Table) for miRNAs were designed in accordance with the work of Chen et al. [29]. Quantitative real-time PCR (qRT-PCR) was conducted with an iCycler iQ Real-Time PCR System (Bio-Rad, USA) and an iQ SYBR Green Supermix Kit (Bio-Rad) in a total volume of 25 μl, including 10 pM of each gene-specific forward and reverse primer and 50 ng of cDNA, which were added into each tube. The qPCR reaction program was set as follows: 94°C for 2 min, followed by 35 cycles of 94°C for 30 seconds, an optimized annealing temperature for 1 minute and 72°C for 90 seconds. All qRT-PCR experiments have been done in three biological and three technical replicates. Rice 18S rRNA was used as an internal control (forward primer 5ʹ-ATAACTCGACGGATCGCAAG-3ʹ; reverse primer 5ʹ-CTTGGATGTGGTAGCCGTTT-3ʹ). The differential expression of miRNAs was calculated using Ct and the 2^-ΔΔct^ formula [30]. Finally, student t test was performed for comparison between normal and treatments. A value of p< 0.05 was considered significant.

## Results

Four small RNA libraries were prepared from root tissues, namely, well watered (WW), water-deprived (WD), the water-deprived part of a split-root system (SpWD) and the well-watered part of a split-root system (SpWW). High-throughput sequencing of these samples using Illumina HiSeq2000 revealed over 26 million raw reads for each library. All libraries’ reads were quality-filtered and the remaining clean reads as small RNAs were mapped in the genome. Finally, 51.12%, 30.10%, 26.94% and 19.68% of reads were precisely mapped in the genome for the WW, WD, SpWW and SpWD libraries, respectively (Table 1).

**Table 1:** Summary of total and unique reads in small RNA libraries.

		# Total small RNAs	# Unique small RNAs
	Total reads	29,313,469	
**WW**	Small RNAs	28,469,954	2,109,016
** **	Mapped to genome	23,403,380	1,078,133
	Total reads	26,419,744	
**WD**	Small RNAs	25,048,989	2,680,261
** **	Mapped to genome	16,045,155	806,687
	Total reads	30,314,575	
**SpWW**	Small RNAs	29,340,181	2,004,540
** **	Mapped to genome	21,244,887	540,016
	Total reads	26,779,435	
**SpWD**	Small RNAs	25,931,301	2,161,218
	Mapped to genome	17,579,453	425,265

**Fig 2:**
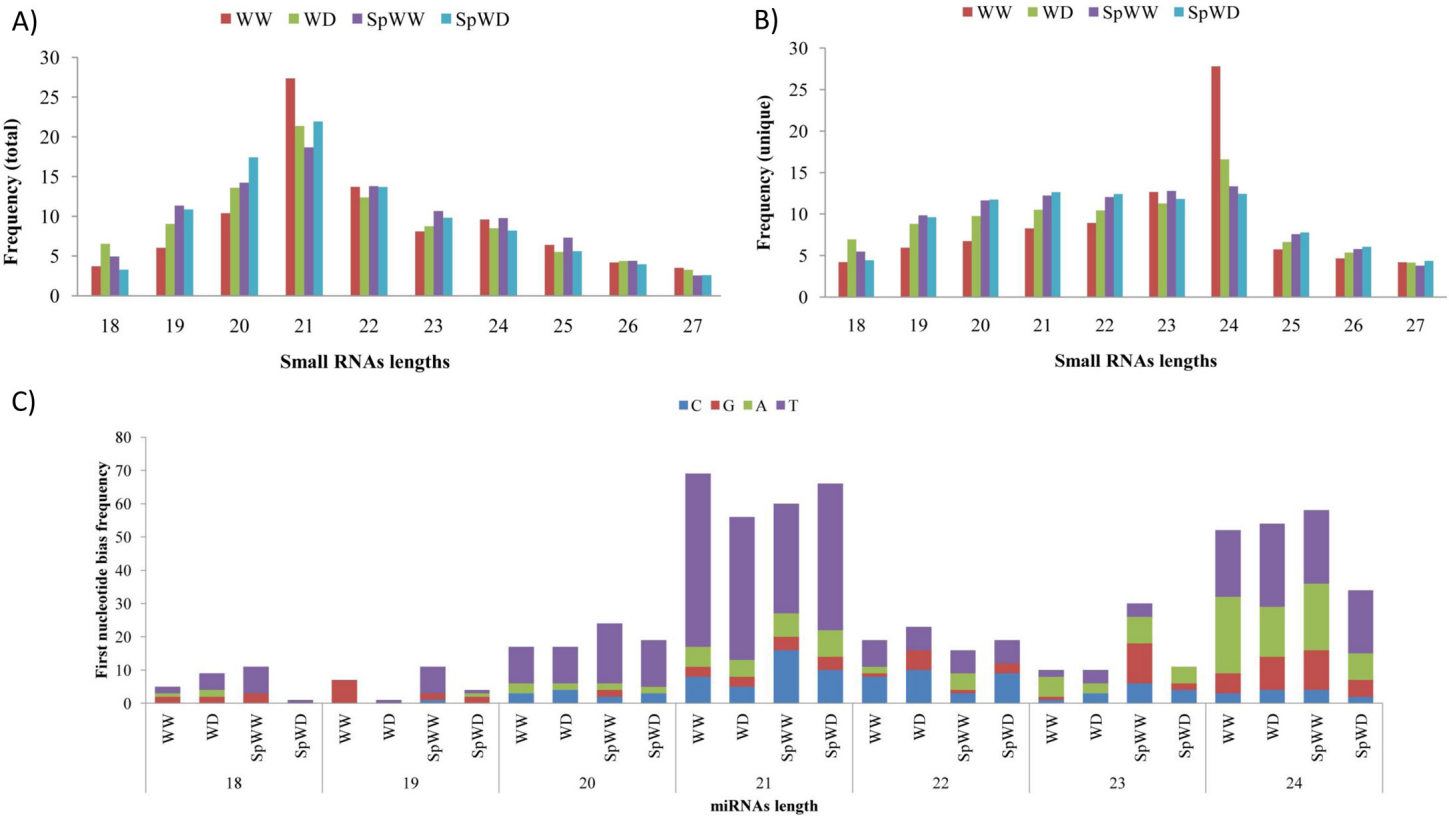
Size distributions of small RNAs and First-nucleotide bias of miRNAs. (A) and (B) The size distribution of both total and unique small RNAs that were identified in all libraries. (C) First-nucleotide bias for 18–24-nt-long miRNAs.

In order to characterize the small RNAs of different libraries, the size distributions of total and unique small RNAs were investigated and are summarized in Fig 2A and 2B. The majority of the total small RNA population was 21 nt in length, and the next largest groups were 20 and 22 nt. Investigating the distribution of unique small RNAs indicates that, most of the unique small RNAs in WW and WD libraries were 24 nt in length. However, unique small RNAs in SpWW and SpWD libraries were in the length range of 20–24 nt. This results are consistent with previous reports on other species, such as *Brassica juncea* and *Citrus aurantifolia* [31,32].

By comparing the total and unique small RNAs to repeat-associated RNAs, GenBank, Rfam, exons and introns of mRNAs and miRBase 17.0, high-throughput sequencing data were classified into 11 groups, namely, rRNA, tRNA, snRNA, snoRNA, repeat, miRNA, intron_sense, intron_antisense, exon_sense, exon_antisense and unannotated reads. The results of the distribution of total small RNAs showed that the proportion of intron_sense declined in the WD, SpWW and SpWD libraries compared with that in the WW library. In addition, rRNAs increased in the SpWD and SpWW libraries compared with those in the WW and WD libraries. The distributions of unique small RNAs in the WD, SpWW and SpWD libraries were approximately similar. This was also true in the case of miRNAs. The percentage of miRNA sequences significantly decreased from 0.2% in the WW library, to 0.13% in WD and 0.09% in the SpWW and SpWD libraries (S1 Fig).

The most abundant miRNAs in each members of a miRNA family were used for analyzing nucleotide bias in different conditions. We observed first nucleotide bias in 21-nt-long miRNAs towards uracil, while uracil and adenine were the two major nucleotides in 24-nt-long miRNAs (Fig 2C).

### Known miRNAs

Mature miRNAs are processed from precursors with an imperfectly paired hairpin which are generated from the transcription of MIR genes by RNA polymerase II [7]. Some of the miRNA reads matched perfectly with more than one precursor. In these cases, the names of the different members were integrated into a single term and all repetitive reads were removed, for example, miR167a, miR167b and miR167c were named miR167a-c, with an average absolute count of 796 in the WW library. In this study, we detected 10,671 unique miRNA reads (S2 Table) that belong to 394 of the 491 registered miRNAs in miRBase version 17.0. Among the 10,671 unique identified miRNA reads, 7102, 5487, 2729 and 2977 reads were detected in the WW, WD, SpWW and SpWD libraries, respectively. All reads that were mapped to the precursor were separated into two main groups, mature miRNAs that were previously identified as the most abundant miRNAs [19], and isomiRs, which are isoforms of the most abundant miRNAs that are originated from imprecise or alternative cleavage of dicer during pre-miRNA processing [33]. The most abundant miRNAs constituted about 91% of total miRNA reads. We also detected 381, 359, 303 and 311 miRNA family members for the WW, WD, SpWW and SpWD libraries, respectively. A Venn diagram showing the numbers of common and specific miRNA members under different conditions is provided in S2A Fig. Furthermore, in terms of the frequency of miRNAs, miR168a was the most prevalent, followed by miR156a-j and miR166a. The top 40 most abundant expressed miRNAs are shown in S3 Fig. Among these, 16 were exactly the same as miRNAs registered in miRBase and 23 were differed from miRNAs registered in miRBase by one to three nucleotides. MiR444c was one of the “top 40” miRNAs but unlike registered miR444c in miRBase that is generated from 3p arm of stem-loop, this miRNA is generated from 5p arm.

### Novel miRNAs

A characteristic hairpin structure is one of the essential features for predicting novel miRNAs [34]. The presence of a dicer cleavage site and the minimum free energy of the unannotated small RNA tags that could be mapped in the genome were the other parameters used to evaluate candidate miRNAs [24]. We were able to identify a total of 48, 37, 54 and 24 unique novel miRNA precursors in the WW, WD, SpWW and SpWD libraries, respectively. Two novel miRNA precursors named WW-m0010 and WW-m0013 were seen in all conditions (S2B Fig). The rates of miRNA reads matched to these two novel precursors were relatively high in comparison with other novel precursors. We also observed 235, 211, 283 and 166 unique novel miRNA reads in the WW, WD, SpWW and SpWD libraries, respectively, and overall there were 783 unique novel miRNA reads in all libraries (S3 Table). Our investigation for the identification of novel miRNAs that were paralogous to registered miRNAs revealed that NR078, NR076 and NR077 are paralogous to ath-miR156a. Likewise, NR304 and NR694 are paralogous to osa-miR1436 and osa-miR1846e, respectively (S4 Table). In addition, we observed that precursor length varies from 64 to 367 nt in WW, from 66 to 372 nt in WD, from 66 to 345 nt in SpWW and from 68 to 367 nt in the SpWD library. The average precursor lengths were 196.33, 199.70, 166.92 and 192.95 nt for the WW, WD, SpWW and SpWD libraries, respectively (S4 Fig). In addition, the minimum folding free energy of the four libraries ranged from −18.4 to −192.5 kcal/mol with an average of −75.01 in WW; from −19.3 to −184.5 kcal/mol with an average of −76.39 in WD; from −18.5 to −184.5 kcal/mol with an average of −53.02 in SpWW; and from −25.9 to −138.5 kcal/mol with an average of −58.50 in SpWD.

### Expression profiling of miRNAs

**Fig 3:**
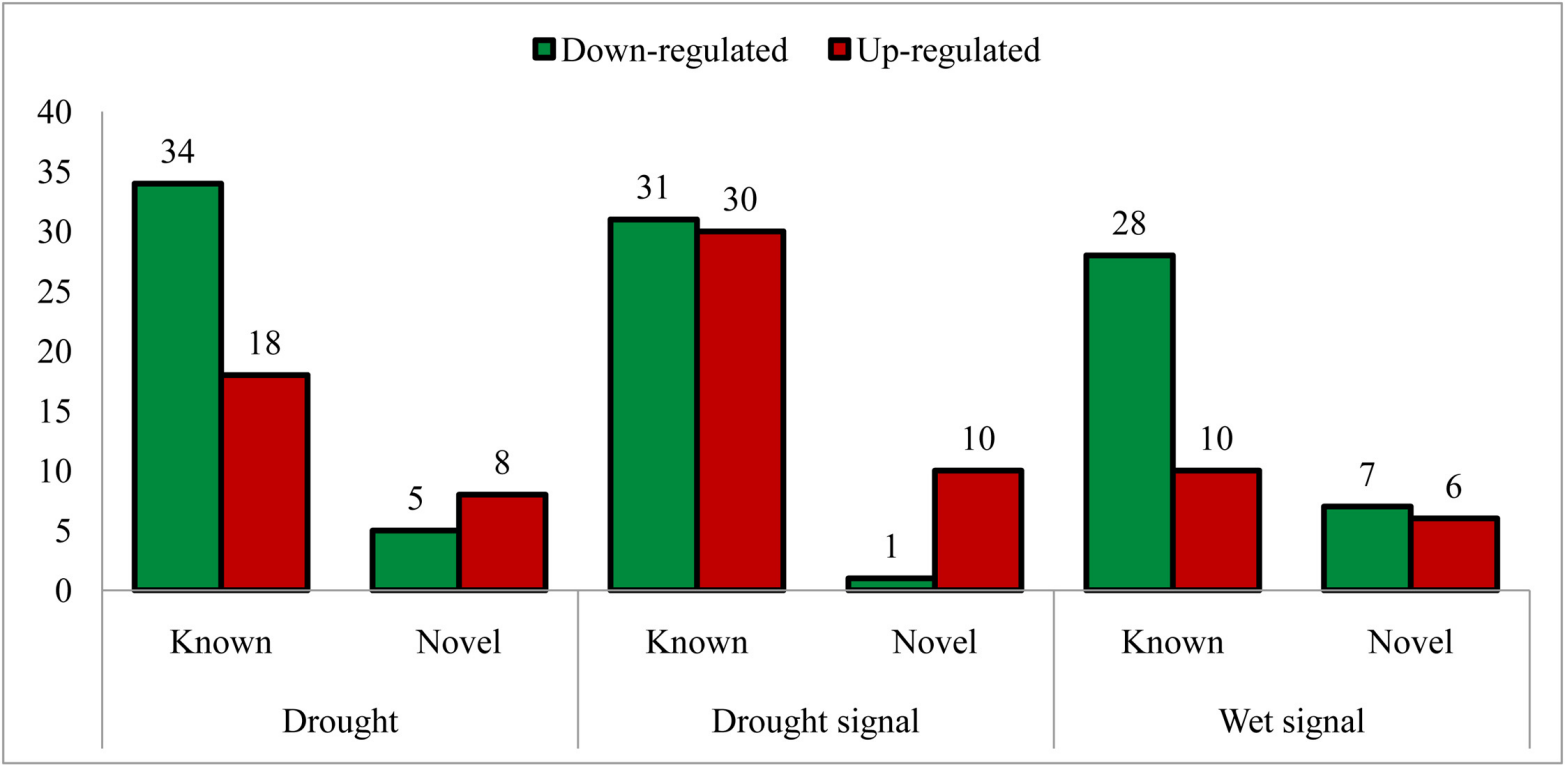
Down-regulated and up-regulated known and novel miRNAs under different conditions. Figure indicates 65,72 and 51 responsive miRNAs under drought stress, drought signaling and wet signaling conditions. As shown in figure, the most numbers of down-regulated and up-regulated miRNAs were observed in drought stress and drought signaling conditions, respectively.

The absolute counts of all miRNAs in this study were normalized to 1 million [a unit of transcripts per million (TPM)]. The normalized counts of each miRNA under the different conditions were compared to determine those miRNAs that were down-regulated and up-regulated under various conditions. Applying the criteria to detect miRNAs with altered expression, 99 miRNAs showed altered expression in at least one comparison (WD/WW, SpWW/WW, SpWD/WW, SpWW/WD, SpWD/WD and SpWD/SpWW) (S5 Table). In this study, we were interested in detecting miRNAs involved in conditions of drought stress, drought signaling and wet signaling. To this end, the WD library was compared with the WW library, which led to the identification of 65 (52 known and 13 novel) drought-responsive miRNAs. The well-watered part of the split-root system (SpWW) could receive drought signals from the water-deprived part (SpWD). Thus, comparing SpWW to WW is useful for identifying miRNAs involved in conditions of drought signaling. By comparing these two libraries, we identified 72 (61 known and 11 novel) drought-signaling-responsive miRNAs. In addition, the water-deprived part of the split-root system (SpWD) could receive wet signals from the well-watered part (SpWW). Therefore, comparison between SpWD and WD could aid the identification of wet-signaling-responsive miRNAs. In this comparison, we identified 51 (38 known and 13 novel) wet-signaling-responsive miRNAs (Fig 3). Overall, 92 and 22 of the known and novel miRNAs showed altered expression in at least one of these three comparisons (S5 and S6 Tables). A fold-change diagram of the known and novel responsive miRNAs is presented in S5 Fig. We observed that, under drought conditions, large numbers of responsive miRNAs were down-regulated. The proportion of responsive miRNAs under drought signaling was greater than under drought stress, which emphasized the role of the miRNA regulatory system in drought signaling. Conversely, we observed the smallest number of differentially expressed miRNAs under wet signaling condition (Fig 3).

**Fig 4:**
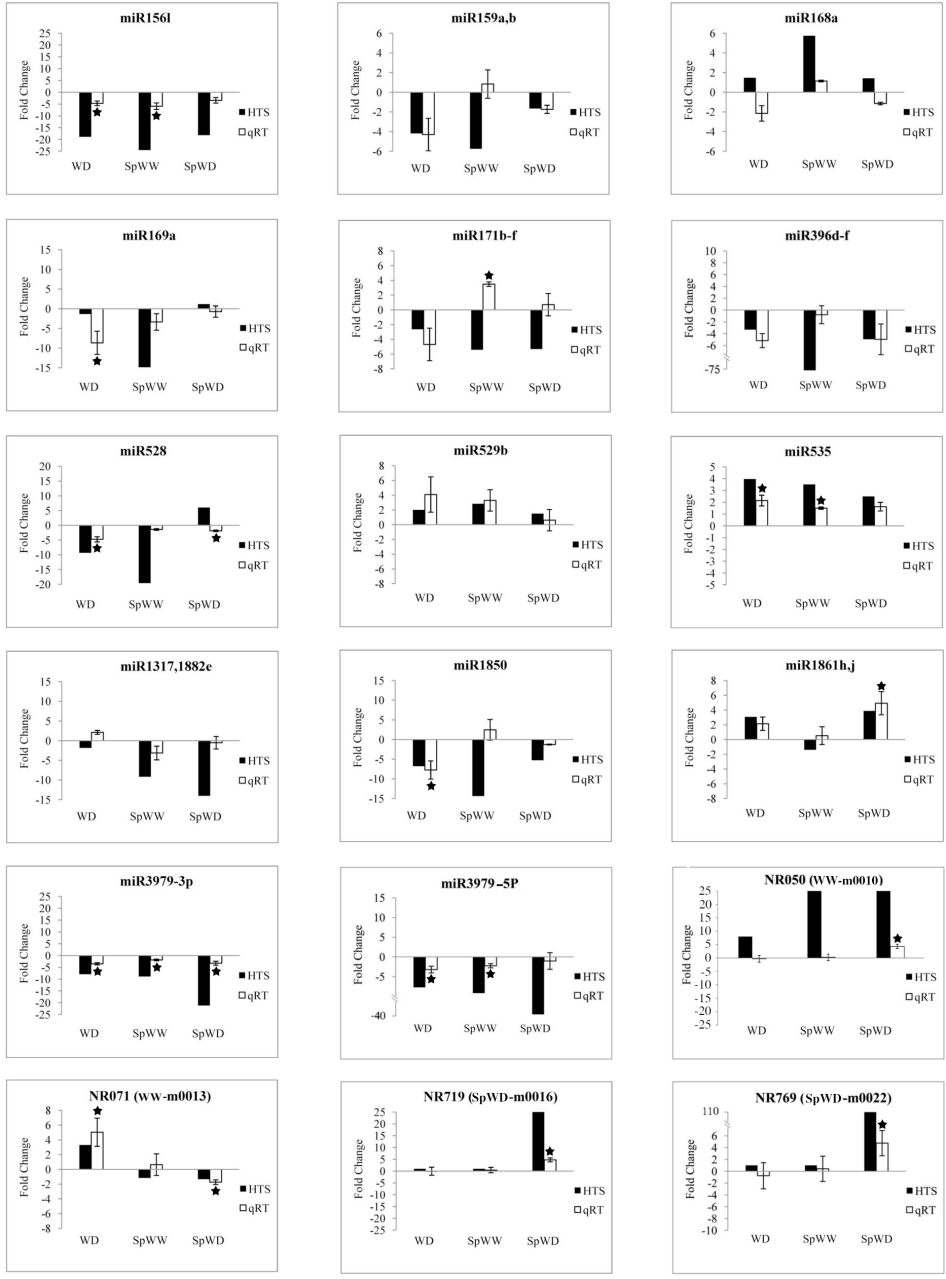
Confirmation of high-throughput sequencing results using qRT-PCR. MiRNA relative expression evaluated by qRT-PCR for WD (WD/WW), SpWW (SpWW/WW) and SpWD (SpWD/WW) compared with the high-throughput sequencing (HTS) results. In this test, 14 and 4 randomly selected (among conserved miRNAs and miRNAs with important targets) known and novel miRNAs have been examined. Asterisk indicates significant differentially expressed miRNAs evaluated by qRT-PCR using student t test. In addition, significant differentially expressed miRNAs evaluated by HTS are presented in S5 Table and S6 Table.

Among these responsive miRNAs, we randomly chose 18 miRNAs for qRT-PCR analysis and compared their expression with that in the well-watered condition (WW) in order to confirm their expression pattern. The results of qRT-PCR indicated that the expression pattern of these miRNAs were similar to the deep sequencing results like miR156l, miR159a,b (for WD and SpWD), miR169a, miR396d-f, miR529b, miR535 and miR3979. However, some inconsistencies were observed like miR168a (for WD and SpWD), miR171b-f (for SpWW and SpWD), and miR528 (for SpWD) (Fig 4). Such inconsistencies between qRT-PCR and deep sequencing results were also observed in previous studies. For example, Li *et al*. observed down-regulation of miR393a by deep sequencing, but conversely this miRNA showed up-regulation by qRT-PCR under drought stress [35]. A similar inconsistency was also found for miR171d [36].

### Target prediction and ontology

**Fig 5:**
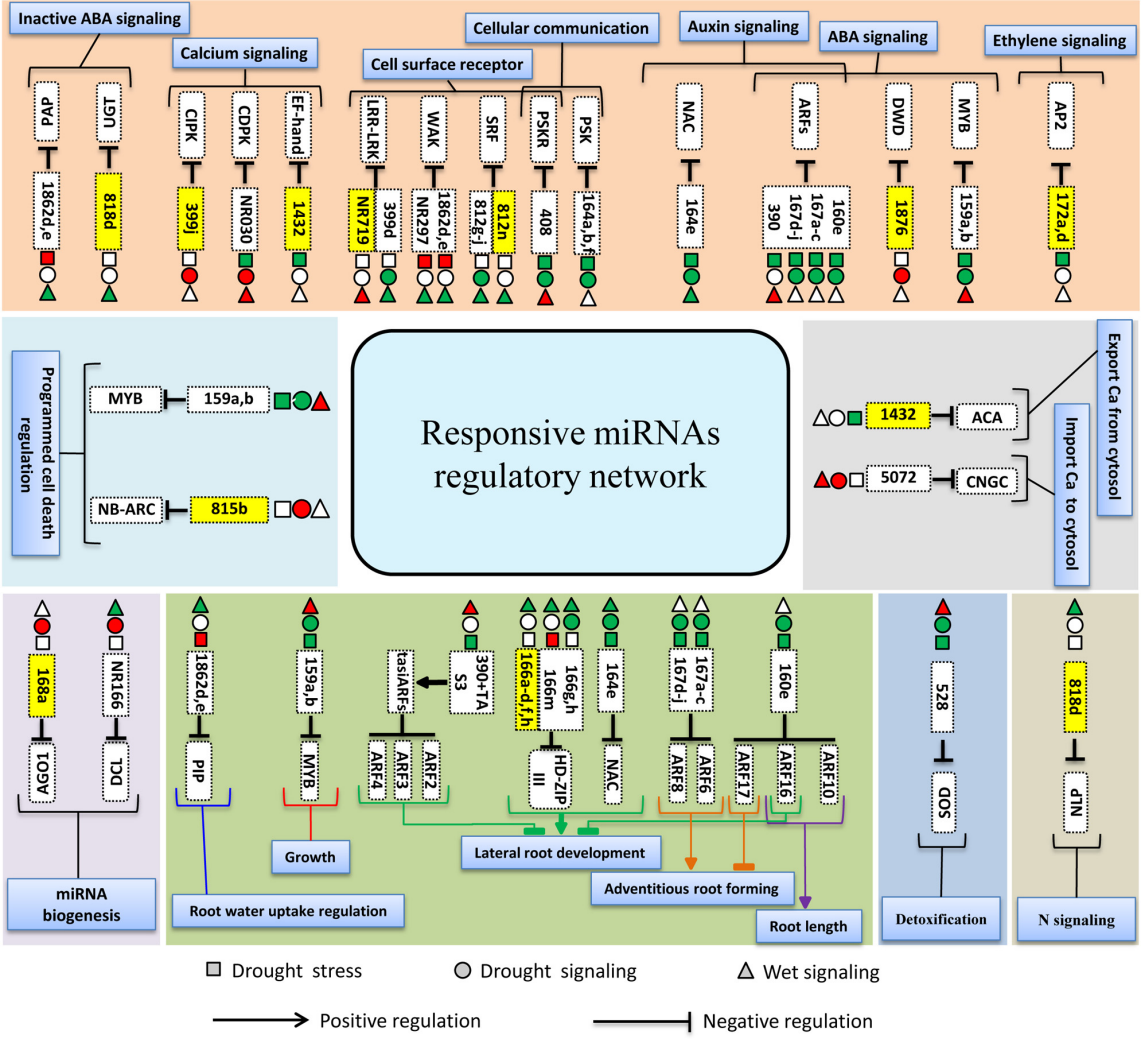
Some of the important responsive known and novel miRNAs and their putative target genes. Red and green indicate up-regulated and down-regulated miRNAs, respectively. MiRNAs highlighted in yellow are those that showed differential expression only under one of drought, drought signaling or wet signaling conditions. In this figure, important miRNAs are categorized according to important pathways that miRNAs and their targets could be involved including detoxification, growth and development, miRNA biogenesis, programmed cell death and signaling pathways.

High complementarity of plant miRNAs and their targets allows us to effectively predict targets [37]. Target prediction of miRNAs enables better understanding of their biological roles. To identify putative targets of the miRNAs, all known and novel miRNAs were used as queries for searches against rice genomic sequences in the plant small RNA target database (psRNATarget). In total, 244, 341 and 239 unique target genes were predicted to be targets of known and novel miRNAs that were responsive to conditions of drought, drought signaling and wet signaling, respectively (S7 and S8 Tables). We predicted many important target genes, such as Auxin-responsive factors (ARFs), APETALA2 (AP2), NAC domain-containing protein, PINHEAD protein, and HD-ZIP III, and numerous other important genes and transcription factors that could be regulated by the miRNAs that showed differential expression under different conditions. Among them regulation of ARF (miR160 and miR167), MYB (miR159), AP2 (miR172), HD-Zip III (miR166) and NAC (miR164) were confirmed experimentally [38,39,40,41,42]. We searched for any miRNA targets that have already been approved by starBase Database [26] and previous published degradom sequencing [27,28]. About 25% of miRNAs predicted targets were confirmed through this comparison (S7 Table). Some of the most important differentially expressed miRNAs and their target genes are shown in Fig 5. In this study, we observed that some miRNAs could regulate multiple target genes. As an example, miR818d could regulate UDP-glucosyl transferase (UGT) and NIN-like protein (NLP) simultaneously. It is important to note that, in most of the cases, different members of a miRNA family can regulate a gene family. For example, class III HD-Zip could be regulated by different members of miR166, including miR166m, miR166g/h and miR166a-d/f/h, although in some cases, their expression patterns were different from each other under different conditions. It was also observed that a single target gene could be regulated by members of different miRNA families. As an example, wall-associated kinase (WAKs) could be regulated by miR1862d/e and NR297. In addition, miRNA target prediction using psRNATarget indicated that most of the miRNA targets (85.39%) could be regulated through cleavage while the other could be inhibited through translation (S7 and S8 Tables).

**Fig 6:**
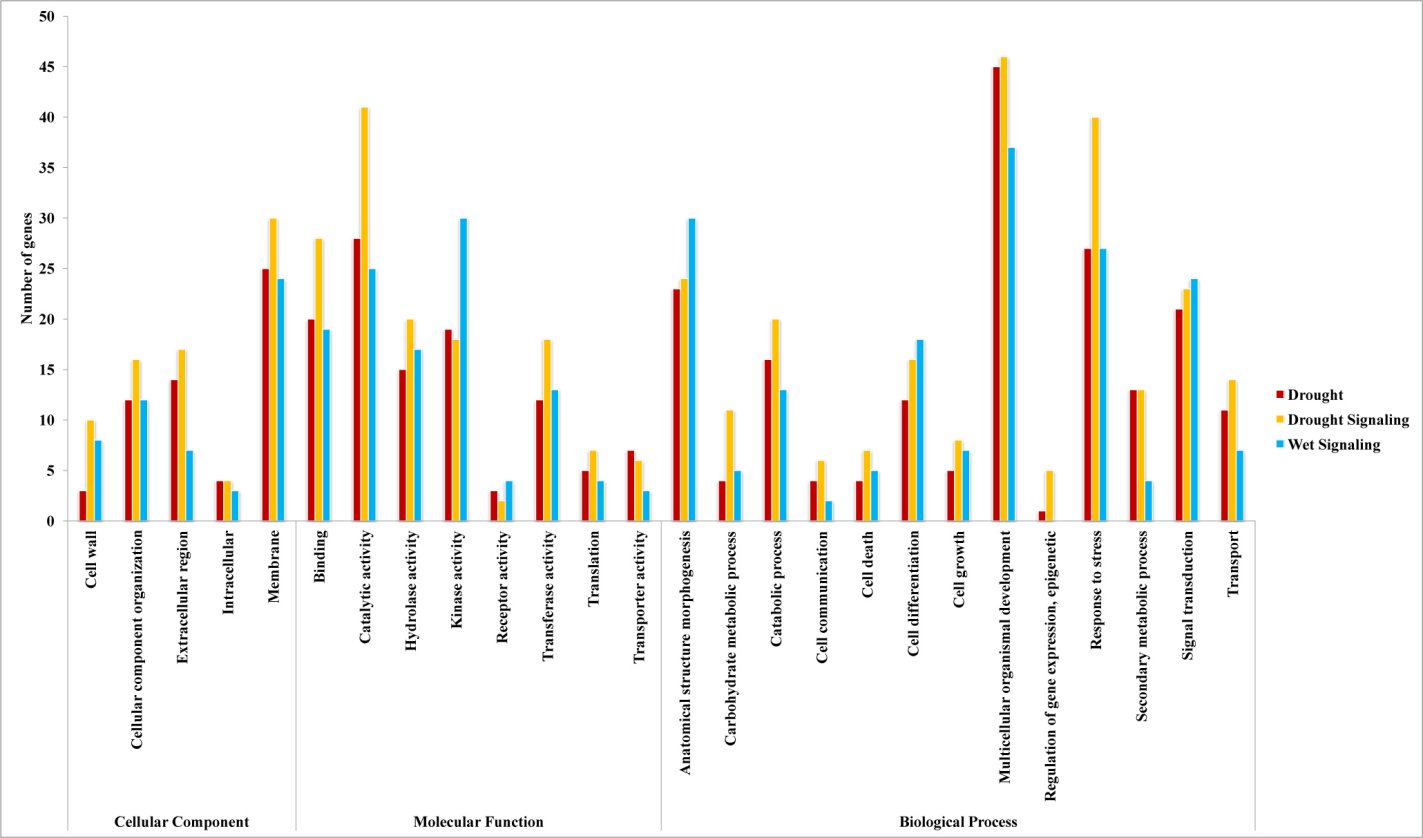
Putative enriched pathways of targets under drought stress, drought signaling and wet signaling conditions.

Gene ontology analysis indicated that drought-stress-responsive miRNAs could play an important role in biological processes. Compared with those which were up-regulated under drought stress, down-regulated miRNAs could regulate a greater proportion of target genes involved in biological processes. For example, miR1850, miR396d-f, miR528 and miR529a are some of the down-regulated miRNAs that could play important roles in the responses to abiotic stimuli. Likewise, miR159a/b are down-regulated miRNAs involved in the regulation of cell death. Under drought-signaling conditions, miRNA target genes were more enriched in a few pathways than in other conditions. The proportion of putative targets of wet-signaling-responsive miRNAs in most of the pathways was lower than drought signaling and drought stress conditions. However, its proportion in signal transduction, cell differentiation, kinase activity and anatomical structure morphogenesis pathways was higher than drought signaling and drought stress conditions (Fig 6).

In this study, we identified 16, 23 and 13 miRNAs that were differentially expressed only under conditions of drought, drought signaling or wet signaling, respectively. Large numbers of these miRNAs were either specific to rice or only present in other species in divergent forms. We were also interested to find putative enriched pathways for these specific miRNAs under different conditions. The results indicated that putative targets of the miRNAs specifically responsive to drought conditions are more involved in secondary metabolic processes. As an example, secondary metabolic processes may be mainly regulated by miR397a under drought stress. Laccase was shown to be a putative target of miR397a. MiR397a and laccase play important roles in lignin biosynthesis [43]. It has also been shown that laccase is involved in *Arabidopsis* root elongation [44]. Thus, miR397a could play important role in lignin biosynthesis and root elongation through regulation of laccase. Other examples we can refer to are miR172a/d and miR1318/1432, which showed differential expression only under drought stress. MiR172a/d can regulate the AP2 transcription factor. It has been shown that AP2 plays an important role in ethylene signaling [45]. MiR1318/1432 can regulate Ca^2+^-transporting ATPase (ACA) and Ef-hand-containing proteins, which have been shown to be involved in the regulation of cytosolic Ca^2+^ concentration and calcium signaling, respectively [46].

**Fig 7:**
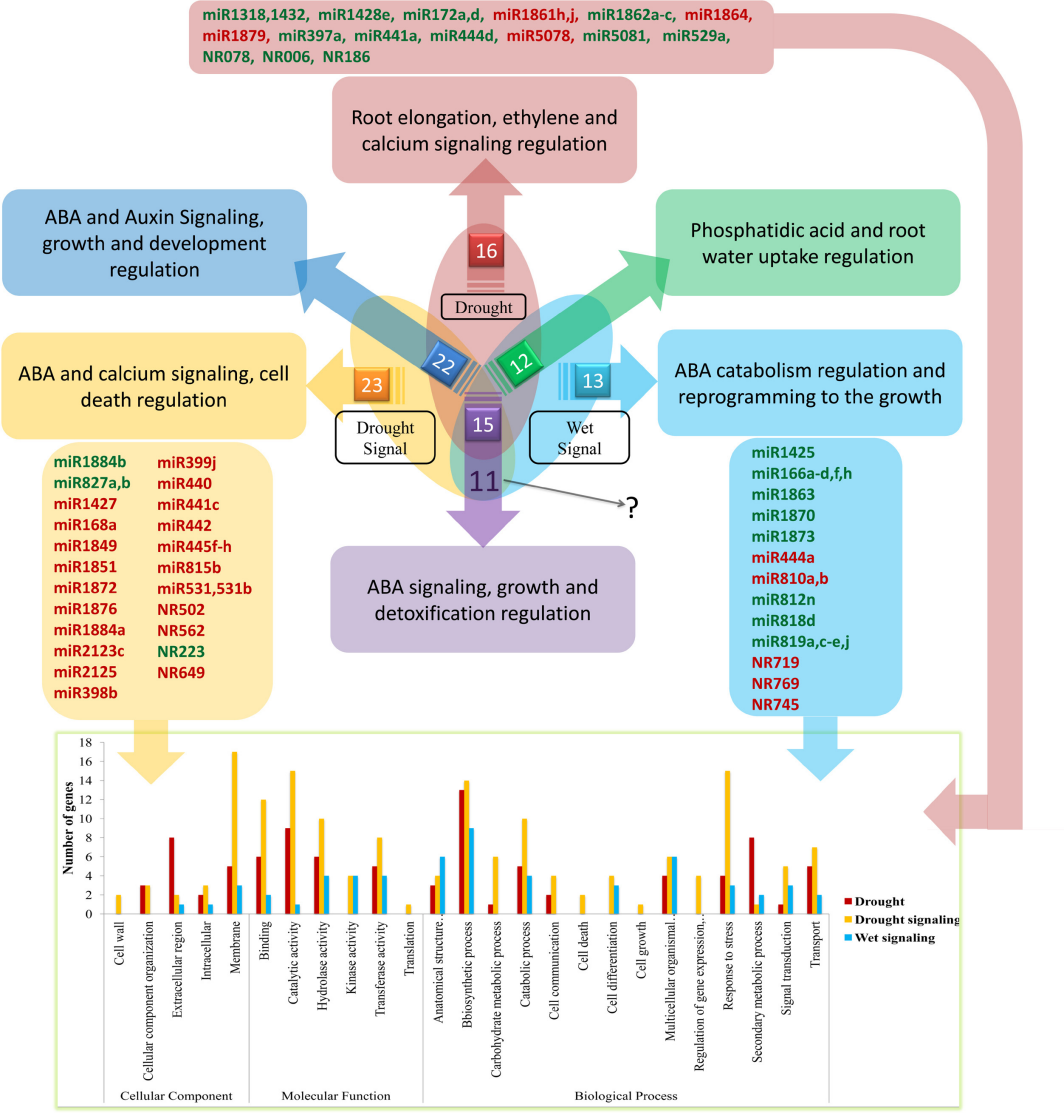
Venn diagram indicating common and specific responsive miRNAs under different conditions. Derived arrows from the Venn diagram refer to prominent enriched pathways of miRNA target genes which specifically or commonly expressed differentially under different conditions. Red and green shading indicates up-regulated and down-regulated miRNAs, respectively. The bar chart indicates enriched pathways for putative target genes of specific responsive miRNAs.

In contrast to what was explained about drought stress above, miRNAs that were specifically responsive under drought signaling conditions are more involved in responses to stress than other two conditions. According to gene ontology results, miR815b is one of these specific miRNAs involved in drought signaling response and can regulate NB-ARC domain-containing protein. It has been shown that the NB-ARC domain is an essential domain of many resistance proteins [47]. MiR1876 and miR399j are two other prominent miRNAs involved in drought signaling response and could regulate WD40 repeat–containing protein (DWD) and CBL-interacting protein kinase (CIPK), respectively. It has been shown that DWD plays a negative role in ABA signaling [48] and CIPK is involved in calcium signaling [46]. In terms of the third different condition of water availability, wet-signaling-specific responsive miRNAs are more involved in anatomical structure morphogenesis than drought stress and drought signaling conditions. For example, miR166a is one of the miRNAs specifically responsive to wet signaling, and it can regulate HD-ZIP III. It has been shown that HD-ZIP III plays an important role in root morphogenesis [49]. MiR818d is another example of miRNA specifically responsive to wet signaling conditions and can regulate UGT. UGT has been shown to be involved in ABA catabolism [50]. Among miRNAs which were responsive to both drought and drought signaling conditions, we observed the prominent roles of these in ABA and auxin signaling, such as miR160 [6]. In addition, we observed 15 miRNAs that were differentially expressed in all conditions. MiR159a/b is one of these miRNAs and it has been shown to be involved in ABA signaling [6]. However, its expression pattern under the drought and drought signaling conditions was not similar to its expression pattern under wet signaling conditions. Finally, among the miRNAs differentially expressed in wet and drought stress conditions, we observed miRNAs like miR1862d/e, which can regulate phosphatidic acid phosphatase (PAP) and aquaporin PIP involved in ABA signaling [51] and root water uptake [52], respectively. The prominent roles of these specific and common miRNAs under different conditions are shown in Fig 7.

## Discussion

Drought stress is the most important abiotic stress, affecting approximately 20% of the total area on which rice is cultivated, globally [53]. Although the roots are the main plant organ involved in water uptake and the first organ that perceives drought stress, little information on drought-responsive miRNAs in rice roots is available. In this study, we applied a partial root drying system along with using fully-droughted and fully-watered plants in order to identify miRNAs involved in conditions of drought stress, drought signaling and wet signaling.

The results of high-throughput deep sequencing indicated that the total number of miRNAs in stress conditions (WD, SpWW and SpWD libraries) were lower than in normal conditions (WW), which suggests the presence of a mechanism to reduce total number of miRNAs under stress. Although the frequencies of unique small RNAs of the SpWW and SpWD libraries were found to be similar, but the frequencies of total small RNAs were not alike (S1 Fig). Likewise, the SpWW and SpWD libraries were similar in terms of their numbers of unique miRNAs, although their total numbers of miRNAs differed. Thus, probably plant produces similar types of small RNAs but with different abundances in well-watered (SpWW) and water-deprived parts (SpWD) of roots that are attached to the same shoot. These results show the important role of regulatory networks that can decrease or increase the abundance of each miRNA in different conditions and consequently change the expression of the miRNAs’ target genes.

In contrast to miR169 being the most abundant miRNA in rice as reported by Sunker et al. [54], in our study, miR168a was the most abundant. Specifically, miR168a was detected 1,058,445 times (664,336 TPM) in the WW library and was the only expressed member of the miR168 family, which represented about 20% of each library. Another member of this family, miR168b, was not detected. Expression pattern results of both miR168a and miR168b in this study is consistent with previous researches [54,55]. Furthermore, Vaucheret reported that miR168a is expressed at a high level and predominantly produces 21-nt-long miRNAs, but conversely, miR168b is expressed at a lower level and produces 21- and 22-nt-long miRNAs in equal amounts [56]. Likewise, in our study, the most common product of miR168a was 21 nt long. It has been shown that miR168a plays important roles in plant development and provides a layer of adaptive responses to endogenous or environmental fluctuations [56].

In this study, we identified 52 known drought stress-responsive miRNAs, some of which have been reported previously (Table 2). Expression patterns of responsive miRNAs were not same as previous reports in some cases. This inconsistency may have been due to differences in the duration of stress, developmental stage, stress severity, growth conditions, tissues used and methods used for studying the differential expression [57]. For example, miR1318/1432 showed an expression pattern that differed from a previous report [58], probably because of differences in the species used, duration of stress, stress intensity and method employed. Furthermore, in our results, a great number of drought-responsive miRNAs were down-regulated (34 out of 52 known drought-responsive miRNAs). Shuai *et al*. also observed extensive down-regulation of drought-responsive miRNAs in *Populus trichocarpa* [59]. Target prediction analysis indicated that these down-regulated miRNAs could regulate a large number of targets, suggesting the importance of a positive regulatory system along with a negative regulatory system in response to drought stress.

**Table 2:** Drought-responsive miRNAs identified in this study that were also detected in previous studies.

miRNAs	Data published elsewhere			Refs
miR1318,1432 (↓)	*T*. *dicoccoides*	miR1432 (↑)	AUCAGGAGAGAUGACACCGAC	[58]
miR1428e (↓)	*O*. *sativa*	miR1428e (↓)	AAUUCACAGGCCCUAUCUUGUG	[19]
miR159a,b (↓)	*P*. *vulgaris*	miR159a.2 (↑)	CUUCCAUAUCUGGGGAGCUUC	[83]
miR160e (↓)	*P*. *euphratica*	miR160e (↑)	UGCCUGGCUCCCUGAAUGCCA	[36]
miR164a,b,f (↓)	*P*. *euphratica*	miR164a (↓)	UGGAGAAGCAGGGCACGUGCA	[36]
miR164d (↓)	*P*. *euphratica*	miR164d (↓)	UGGAGAAGCAGGGCACGUGCU	[36]
miR164e (↓)	*P*. *euphratica*	miR164e (↓)	UGGAGAAGCAGGGCACGUGAG	[36]
miR166m (↑)	*P*. *euphratica*	miR166m (↓)	UCGGACCAGGCUUCAUUCCCU	[36]
miR167a-c (↓)	*P*. *euphratica*	miR167a (↑)	UGAAGCUGCCAGCAUGAUCUAA	[36]
miR167d-j (↓)	*A*. *thaliana*	miR167d (↓)	UGAAGCUGCCAGCAUGAUCUGG	[69]
miR169b,c (↓)	*O*. *sativa*	miR169b (↑)	CAGCCAAGGAUGACUUGCCGG	[36]
miR171b-f (↓)	*P*. *euphratica*	miR171d (↑)	UUGAGCCGCGCCAAUAUCAC	[36]
miR172a,d (↓)	*P*. *euphratica*	miR172d (↓)	GGAAUCUUGAUGAUGCUGCAU	[36]
miR1850 (↓)	*B*. *distachyon*	miR1850 (↑)	UGGAAAGUUGGGAGAUUGGGG	[71]
miR390 (↓)	*B*. *distachyon*	miR390 (↑)	AAGCUCAGGAGGGAUAGCGCC	[71]
miR396d-f (↓)	*P*. *euphratica*	miR396f (↑)	UCUCCACAGGCUUUCUUGAACU	[36]
miR397a (↓)	*P*. *euphratica*	miR397a (↑)	UCAUUGAGUGCAGCGUUGAUGU	[36]
miR397b (↓)	*P*. *euphratica*	miR397b (↑)	UUAUUGAGUGCAGCGUUGAUG	[36]
miR408 (↓)	*P*. *euphratica*	miR408 (↑)	AUGCACUGCCUCUUCCCUGGC	[36]
miR528 (↓)	*B*. *distachyon*	miR528 (↑)	UGGAAGGGGCAUGCAGAGGAG	[71]
miR529a (↓)	*O*. *sativa*	miR529a (↓)	CGAAGAGAGAGAGCACAGCCC	[14]

### Potential functions of responsive miRNAs

After identifying potential target genes of the miRNAs differentially expressed under conditions of different water availability, we investigated their functions in cells. We observed several miRNAs with prominent roles, such as miR160, mir159, miR528 and miR172, which can regulate important transcription factors and genes under different conditions (Fig 5). These miRNAs could be involved in ABA signaling, detoxification, growth and many other important pathways, as discussed below.

### Cell signaling regulation under different conditions

We observed several responsive miRNAs that could be involved in the regulation of cell surface receptors, which play roles in the transmission of signals across membranes. Leucine-rich repeat receptor-like protein kinase (LRR-LRK), WAK, strubbelig receptor family (SRF) and, phytosulfokine receptor (PSKR) are cell surface receptor kinases that can be regulated by miRNAs and most of these miRNAs showed differential expression under wet signaling condition. Specifically, miR812n and NR719 are two miRNAs that showed differential expression only under wet signaling condition and they can regulate SRF and LRR-RLK, respectively. Thus, these miRNAs could play important roles in the regulation of wet signal perception. In addition, miR408 and miR164a/b/f are two drought responsive miRNAs that could be involved in cell communication under conditions of both drought stress and drought signaling. These two miRNAs could regulate cell-to-cell communication through the regulation of phytosulfokine (PSK) and phytosulfokine receptor (PSKR) cooperation. PSKR is an LRR-RK, a receptor kinase that can bind to PSK [60]. PSK is a signal molecule that is essential for cell-to-cell communication [61]. Down-regulation of both miR408 and miR164a/b/f could possibly cause higher activities of PSK and PSKR in cell-to-cell communication under drought stress and drought signaling conditions.

In this study, we observed several miRNAs that may play roles in the regulation of Ca^2+^ transporters and sensors under different conditions. Membranes have several Ca^2+^ transporters to export and import this ion [46]. ACA and cyclic nucleotide-gated channels (CNGC) are two important Ca^2+^ transporters that have been shown to be involved in exporting/importing Ca^2+^ from/into the cytosol, respectively [46]. Down regulation of miR1318/1432 under drought stress, probably increase the activity of ACA followed by the export of Ca^2+^ from the cytosol. In addition, miR5072, which was up-regulated under drought signaling and wet signaling conditions could lead to reduction of CNGC activity followed by reduction of Ca^2+^ import into the cytosol. Since maintenance of the Ca^2+^ concentration at a low level is necessary for sufficient cell signaling [62], there is probably a general program in the cell for maintaining Ca^2+^ at a low level under different conditions through miRNA activity.

There are also several Ca^2+^ sensors that are involved in calcium signaling and act as secondary messengers [46]. In this context, CDPK, CIPK and EF-hand family proteins are important Ca^2+^ sensors that could be regulated by NR030, miR399j and miR1318/1432, respectively. NR030, which is involved in regulating CDPK, showed down-regulation under conditions of drought stress. Thus, over-expression of CDPK probably occurs under drought stress. CDPKs are known to play a positive role in the response to abiotic stresses by inducing the expression of stress-responsive genes [46]. It has also been shown that over-expression of CDPK in rice causes over-expression of drought-responsive genes and an increased tolerance to drought stress [63]. In addition, miR1318/1432, which was down-regulated under drought stress condition could also regulate the EF-hand family proteins. Possession of an EF-hand is a characteristic of Ca^2+^ sensors like CDPK [46]. Therefore, down-regulation of miR1318/1432 and NR030 that are involved in regulating Ca^2+^ sensors under drought stress could increase activity of Ca^2+^ sensors followed by increased expression of stress-responsive genes. However, under drought signaling condition, we can probably observe the opposite case, wherein NR030 and miR399j, which could regulate CDPK and CIPK, are up-regulated. Therefore, reduction in the expression of Ca^2+^ sensors followed by reduction in expression of stress-responsive genes could be observed under drought signaling. In contrast to drought stress and drought signaling conditions mentioned above, under wet signaling, miR1318/1432 and miR399j did not show differential expression, although NR030 showed up-regulation. Thus, a decrease of calcium signaling could also be expected under wet signaling condition.

We observed that miRNAs involved in phytohormone pathways under drought stress and drought signaling conditions are more differentially expressed than those under wet signaling condition, which can indicate the role of these miRNAs in phytohormone signaling under drought conditions. It should be noted that some of the miRNAs that contribute to ABA signaling, including miR159, miR160 and miR167 [6], showed similar expression patterns under drought stress and drought signaling conditions, which could be due to the presence of the same phytohormone signal in both conditions. Our previous study showed an increase in the level of ABA under conditions of drought stress and drought signaling in rice [22]. In addition, a reduction in the level of auxin under drought stress was also reported [64]. Several studies have reported the differential expression of miRNAs occurred by changes in the level of phytohormones. For instance, it was reported that, as the level of auxin increased, the expression of miR164 and miR167 increased simultaneously; in addition, an increase in the level of ABA is correlated with a reduction in the expression of miR167 [65]. Therefore, down-regulation of miRNAs like miR167 might be due to the reduction of auxin and the increase in the level of ABA under drought stress and drought signaling conditions.

MiR160 has been shown to be involved in ARF regulation and it was also shown that the differential expression of this miRNA could regulate ABA sensitivity, in which the up-regulation of miR160 leads to ABA insensitivity and over-expression of its target gene (ARF 10) caused ABA hypersensitivity [66]. Thus, it can be expected that down-regulation of miR160 under drought stress and drought signaling leads the plant to increase its sensitivity to ABA. In addition, MYB and DWD, which are involved in ABA signaling, could intensify the increase of sensitivity to ABA. MYB and DWD are regulated by miR159a/b and miR1876, respectively. MYB is considered as positive regulator of ABA signaling [67]. Therefore, down-regulation of miR159 might induce ABA signaling under drought stress and drought signaling conditions, but conversely, its up-regulation in the presence of wet signaling might reduce ABA signaling. MiR1876, which showed differential expression only under drought signaling condition, could regulate DWD. Since DWD is a negative regulator of ABA signaling [48], up-regulation of miR1876 may cause reduction of DWD activity and consequently a reduction of its negative role in ABA signaling. Thus, under drought stress and specifically under drought signaling, miRNAs tend to increase ABA signaling but conversely, miRNAs tend to decrease ABA signaling under wet signaling condition. There are also some other findings in our study which show the reduction of ABA signaling under wet signaling condition by miRNAs. We observed that UGT, which is involved in the conjugation of ABA to abscisic acid glucosyl ester (ABA-GE) [50], could be regulated by down-regulation of miR818d in the presence of wet signaling. Down-regulation of miR818d may cause an increase of UGT activity followed by the conjugation of ABA to the ABA-GE inactive form. Thus, miR818d can play an important role in the reduction of ABA level under wet signaling condition. It was also observed that miR1862d/e, which can regulate PAP, was up-regulated under drought stress and down-regulated under wet signaling. PAP is involved in the catabolism of phosphatidic acid (PA) to diacylglycerol (DAG) and consequently the inhibition of ABA signaling [51]. The up-regulation of miR1862d/e under drought stress may cause the reduction of PAP expression, an increase of PA, followed by an increase in ABA signaling, but conversely, down-regulation of this miRNA under wet signaling condition may have reduced both PA activity and ABA signaling. In addition to miRNAs that could be involved in ABA signaling, we observed response of miR172 that could be effective in ethylene signaling. MiR172a/d, which was differentially expressed only under drought stress, was one of the important miRNAs that could regulate AP2. AP2 is from the AP2/ERF superfamily and can bind to cis-responsive elements of genes involved in response to ethylene [45]. The down-regulation of this miRNA can cause the over-expression of AP2 and consequently the over-expression of stress-responsive genes under drought stress. Altogether, these results indicate that the cell is programmed to regulate ABA, ethylene and calcium signaling in different ways under drought stress, drought signaling and wet signaling conditions through miRNA regulation.

### miRNA biogenesis regulation

Our findings showed that one of the miR168a isoforms could regulate the PINHEAD protein. The PINHEAD protein is similar to argonaut1 (AGO1) and there is an overlap in the expression pattern of these two proteins [68]. Although the most abundant read of miR168a did not show differential expression under all conditions, but this isoform of miR168a was up-regulated under drought signaling condition. Moreover, two previous studies reported differential expression of this miRNA under drought stress in rice and *Arabidopsis* [14,69]. In addition, dicer, another important molecule in miRNA biogenesis, can be regulated by NR166 under drought signaling condition. Thus, there is probably a feedback regulatory system for miRNA biogenesis under drought signaling condition.

#### MiR528 could increase detoxification under drought and drought signaling conditions

In this study, we observed that miR528 could possibly be involved in the regulation of superoxide dismutase (SOD). Since SOD has been shown to act as a reactive oxygen species (ROS) scavenger under stress [70], down-regulation of miR528 might result in an increase in SOD under drought conditions. In our previous study, we observed up-regulation of SOD under drought conditions [22]. Interestingly, in this study we observed down-regulation of miR528 under conditions of drought and drought signaling, which suggests a role of miR528 in SOD regulation. Down-regulation of miR528 was also observed in *Zea mays*, *Brachypodium distachyon* and *Triticum dicoccoides* under drought stress [58,71,72]. Taken together, these results highlight the role of miR528 detoxification in conditions of drought stress.

#### Root growth and developmental regulation under different conditions

Under drought stress, the root system architecture changes in a way that improves water absorbance [73]. In this context, it was observed that plants under normal conditions develop their lateral roots, but under drought stress, the formation and development of lateral roots are decreased, which is considered to be an adaptive response to cope with drought stress [74]. Our surveys showed that the differential expression of miRNAs involved in root formation under drought conditions is probably associated with answering this demand. MiR160 is one of the main regulators of root growth and regulates ARF10, ARF16 and ARF17 [75]. It was reported that the up-regulation of miR160 caused the reduction of ARF10 and ARF16, followed by a reduction of root length, but an increase in ARF16 led to a reduction of lateral root growth [76]. On the other hand, ARF17 is known as a negative regulator of adventitious root formation [38]. In this study, the down-regulation of miR160e was observed under conditions of drought stress and drought signaling, but no differential expression under wet signaling conditions was detected for this miRNA. Such differential expression under conditions of drought stress and drought signaling can cause the over-expression of ARF10, ARF16 and ARF17, followed by the reduction of lateral and adventitious root formation. However, in the case of wet signaling conditions, normal growth of lateral roots is expected through the regulatory role of miR160e. Another example for miRNAs which showed importance in root formation is miR167, that showed down-regulation under drought stress and drought signaling conditions. MiR167 regulates ARF6 and ARF8 which play positive roles in the formation of adventitious roots [77]. Thus, the down-regulation of miR167a-c and miR167d-j under drought stress and drought signaling can lead to the formation of adventitious roots. MiR390 is another miRNA that plays a role in regulating ARFs, but unlike miR160 and miR167, it regulates ARFs indirectly. MiR390 causes an increase in *TAS3*-derived trans-acting short-interfering RNAs (tasiARFs) through the cleavage of *TAS3* non-coding RNA [78]. Subsequently, ARF2, ARF3 and ARF4, which inhibit lateral root formation, are suppressed by tasiARFs [78]. Thus, miR390 promotes lateral root formation [78]. In our experiments, miR390 showed down-regulation and up-regulation under conditions of drought stress and wet signaling, respectively. Therefore, while this miRNA probably causes a reduction of lateral root growth under drought stress, in the presence of wet signaling, an increase in such growth is expected. In addition to ARFs that play an important role in root formation, several reports have described the roles of HD-ZIP III and NAC in root formation [49,79]. HD-ZIP III has been shown to play a positive role in lateral root formation [49] and it has been reported that up-regulation of miR166, which can regulate HD-ZIP III, decreases the number of lateral roots [80]. In the present study, miR166m showed up-regulation under drought stress which suggests that, as miR166m is up-regulated, HD-ZIP III is reduced, leading to the reduction of lateral roots under drought stress. However, in the case of wet signaling, miR166g/h, miR166m and miR166a-d/f/h showed down-regulation, which can lead to an increase of HD-ZIP III and consequently an increase in lateral root growth. Under drought signaling, miR166g/h was down-regulated, but miR166a-d/f/h and miR166m did not show any significant differential expression. Thus, this miRNA family (miR166) has a greater tendency to regulate lateral roots under drought stress than under drought signaling. We also observed that miR390 did not show significant differential expression under drought signaling condition. Thus, although in certain cases the tendency to decrease the lateral root growth through miRNA could be detected under drought signaling condition, but it seems that, compared with drought stress, fewer miRNAs tended to reduce lateral root growth. On the other hand, a strong tendency to increase lateral root growth under wet signaling condition through the regulatory role of miRNAs can be inferred.

Since an increase of MYB has a negative role in growth regulation and leads to growth reduction, the role of miR159 in regulating MYB categorizes this miRNA as one of the important miRNAs involved in growth [81]. According to our results, down-regulation of miR159a/b could increase MYB expression followed by growth reduction under conditions of drought and drought signaling. However, in the case of wet signaling, the reduction of MYB followed by an increase in growth is expected. It has also been shown that MYB transcription factors play a positive role in programmed cell death [81]. Thus, the enhancement of cell death under drought and drought signaling conditions and its inhibition under wet signaling are suggested.

MiR818d was among the miRNAs that showed differential expression only under wet signaling. This miRNA could regulate the NLP transcription factors. It has been observed that NLP7 is involved in nitrogen sensing and signaling and also acts as a positive regulator of nitrate-responsive genes, which regulate the growth and metabolism of cells [82]. Thus, the down-regulation of miR818d under wet signaling probably leads to the over-expression of NLP transcription factors and nitrate-responsive genes, which could be effective for growth.

Root water uptake through water channel proteins that are present in membranes is important under drought stress, and aquaporin proteins which are located in the membranes of root cells, play an important role in transferring water to roots [52]. We observed that miR1862d/e, which regulates aquaporin PIP, showed up-regulation under drought stress, but in the presence of wet signaling, they were down-regulated. Thus, these miRNAs probably play negative and positive roles in root water uptake under drought stress and wet signaling conditions, respectively.

## Conclusion

**Fig 8:**
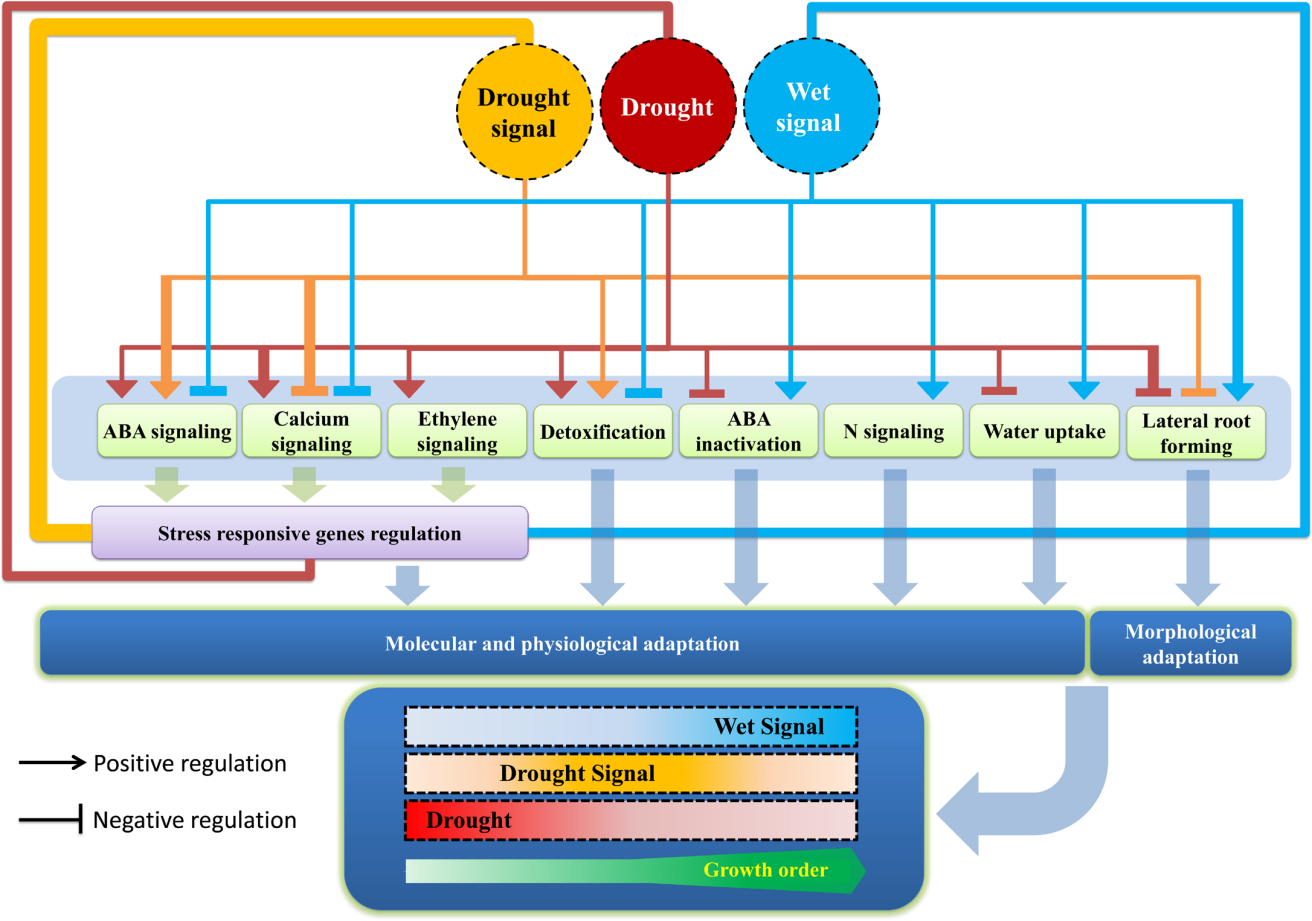
Effects of drought-, drought-signaling- and wet-signaling-responsive miRNAs in different pathways. Thickness of lines indicates the abundance of miRNAs that are involved in each pathway. Figure indicates how responsive miRNAs could be involved in physiological or morphological adaptation and finally how their actions could lead to energy diversion between stress adaptation and growth under drought, drought signaling and wet signaling conditions.

In this study, we observed that different strategies are applied by cells to deal with various levels of water availability. These various strategies could be regulated by miRNAs to maintain the vitality of plants under drought stress, drought signaling and wet signaling conditions. Our results indicate that down-regulation of a large numbers of drought-responsive miRNAs under drought stress probably cause the over-expression of their target genes which are involved in responses to stress, cell death and other biological processes. In addition, our results suggest that the expression of stress-responsive genes could also be induced through calcium, ethylene and ABA signaling under drought stress conditions mediated by miRNA activity. Furthermore, the tendency to decrease lateral root growth through miRNA regulation could occur under drought conditions. Therefore, under drought stress, these drought responsive miRNAs presumably change plant`s strategy to cope with drought stress. Under drought signaling condition, we observed that miRNAs tended to decrease calcium signaling, although ABA signaling was suggested to be induced. In addition, our results indicated that drought-signaling-responsive miRNAs are more involved in the regulation of stress responses than other biological process. Thus, strict control of biological processes by miRNA regulation could occur under conditions of drought signaling. Furthermore, in our previous study partial drying system showed more ABA level than in well-watered condition [22]. We conclude that, when a plant has adequate water but some of its roots are exposed to drought conditions (or when water is available but is inadequate), the plant shifts its biological system from normal to high alert. The plant also establishes a major regulatory network to employ responsive miRNAs in order to prepare itself to endure drought stress. On the other hand, it was observed that wet-signaling-responsive miRNAs could be involved in the morphogenesis of anatomical structures, especially roots. In addition, unlike the cases in drought stress and drought signaling, the miRNA expression patterns suggest that, under wet signaling, miRNAs tend to decrease ABA signaling and increase lateral root development. Thus, under wet signaling, miRNAs tend to regulate biological processes in the opposite way that they do under drought and drought signaling conditions (Fig 8). Therefore, as we observed in our previous study, lower ABA level and more relative water content in partial drying system than fully-droughted [22] could be as result of wet signals action in competition with drought signals that could decrease drought stress intensity. These results indicate that these wet signaling responsive miRNAs can promote the growth of a plant in the presence of wet signaling. This can be followed by the diversion of resources from stress-responsive centers to growth and development centers. Conversely, the presence of drought signaling and especially drought stress leads miRNAs to divert energy away from growth and development centers to stress-responsive ones (Fig 8).

Further functional studies on the miRNAs which showed dynamic expression patterns under different drought conditions can be manipulated in order to achieve certain functional advantages.

## Supporting Information

S1 Fig Distribution of unique and total small RNAs in different libraries. (TIF)

S2 Fig Common and specific detected known (a) and novel (b) miRNAs.
(TIF)

S3 Fig Top 40 most abundant miRNA reads. Y-axis indicates abundant miRNA members and X-axis indicates counts of miRNAs that are changed to logarithmic scale with base 10. Figure indicates that miR168, miR156 and miR166 are most abundant miRNAs.
(TIF)

S4 Fig Length distribution of novel miRNA precursors. (TIF)

S5 Fig Fold-changes of responsive known and novel miRNAs under different conditions. (TIF)

S1 Table Details of the primers used in this study. (XLSX)

S2 Table List of the 10,671 miRNAs detected in this study. (XLSX)

S3 Table List of the 783 novel miRNAs detected in this study. (XLSX)

S4 Table List of detected novel miRNAs Paralogous to miRNAs registered in miRBase. (XLSX)

S5 Table Ninety-nine differentially expressed known miRNAs in all conditions. (XLSX)

S6 Table Twenty-two differentially expressed novel miRNAs in all conditions. (XLSX)

S7 Table Complete list of all differentially expressed known miRNAs and their target genes. (XLSX)

S8 Table Complete list of all differentially expressed novel miRNAs and their target genes. (XLSX)
