# Supplementary figures and images for: MicroRNA Signatures of Drought Signaling in Rice Root
Source: PLoS One. 2016 Jun 8;11(6):e0156814. doi: 10.1371/journal.pone.0156814 (PMC4898717; doi:10.1371/journal.pone.0156814)

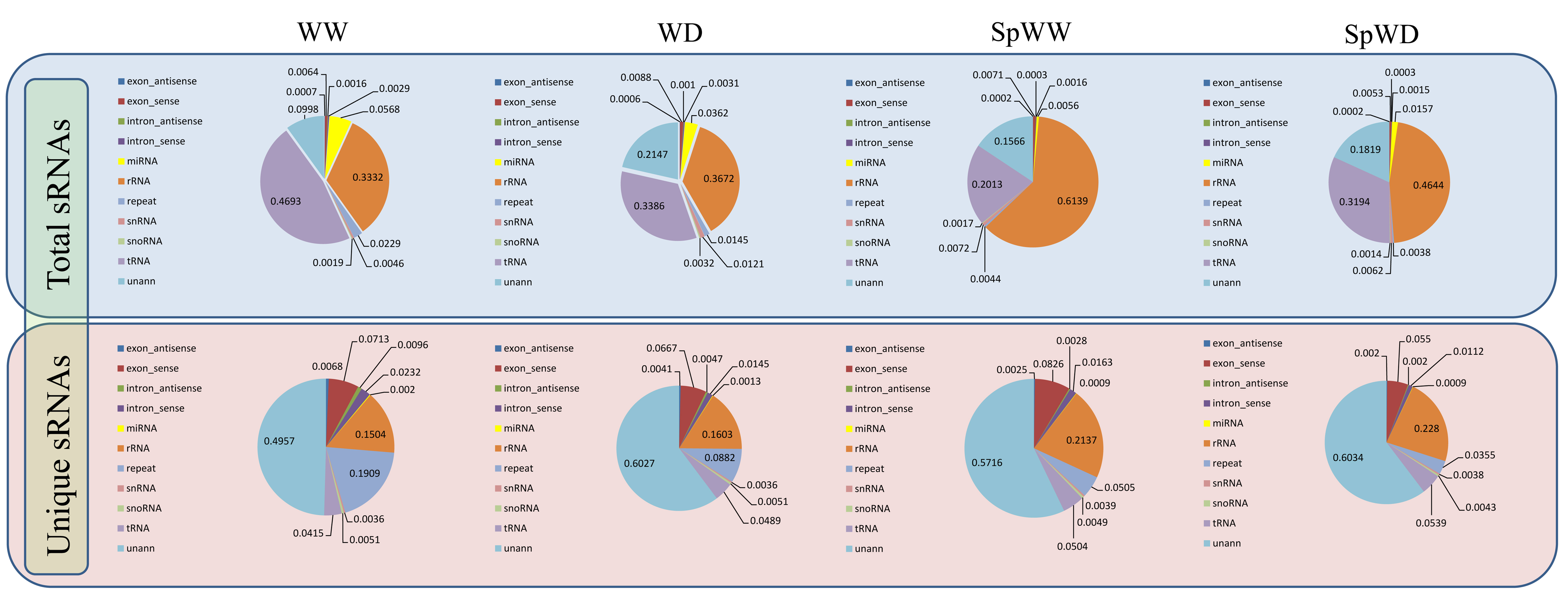

Supplement: S1 Fig — (TIF) [file pone.0156814.s001.tif]

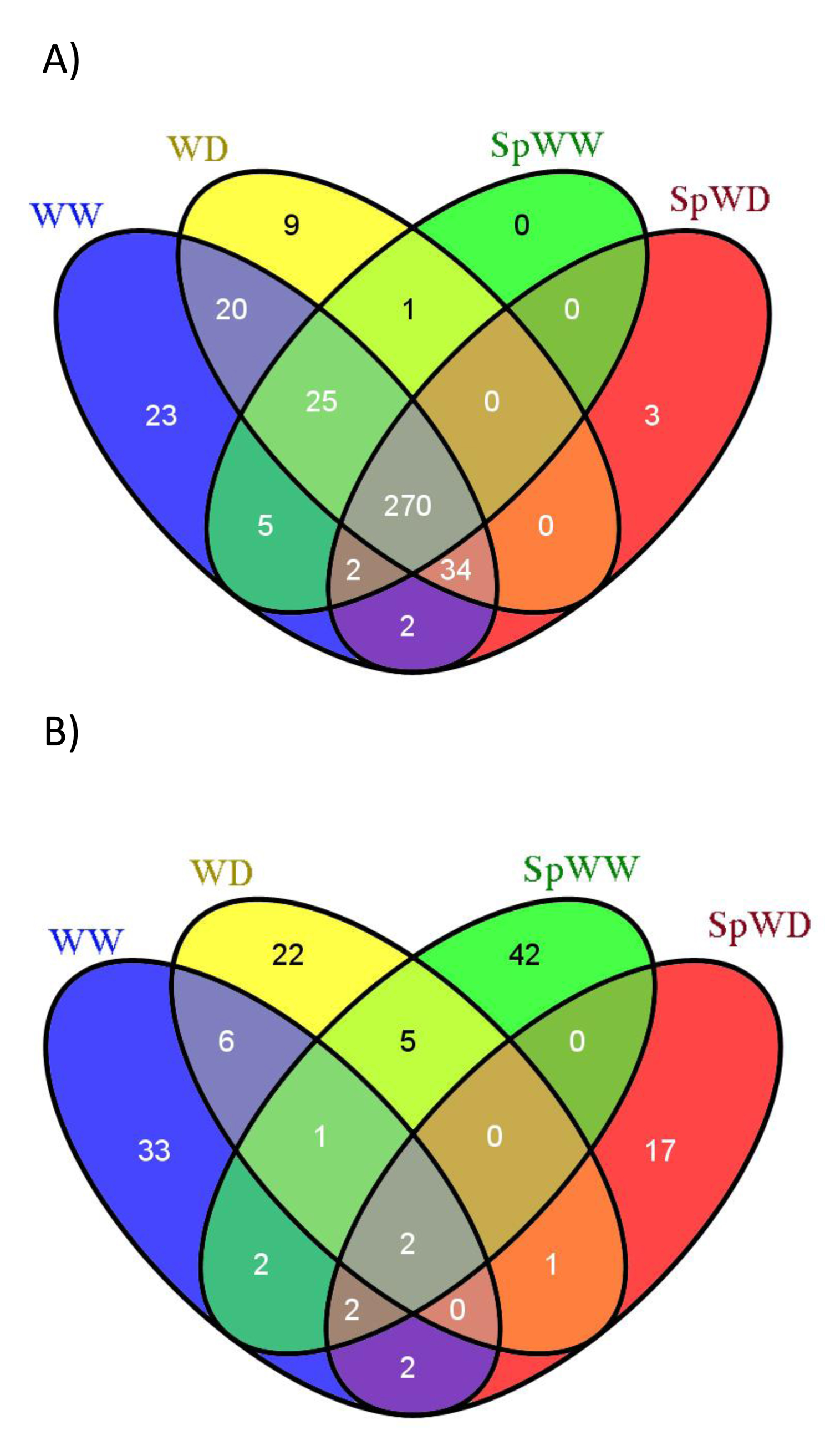

Supplement: S2 Fig — Common and specific detected known (a) and novel (b) miRNAs. (TIF) [file pone.0156814.s002.tif]

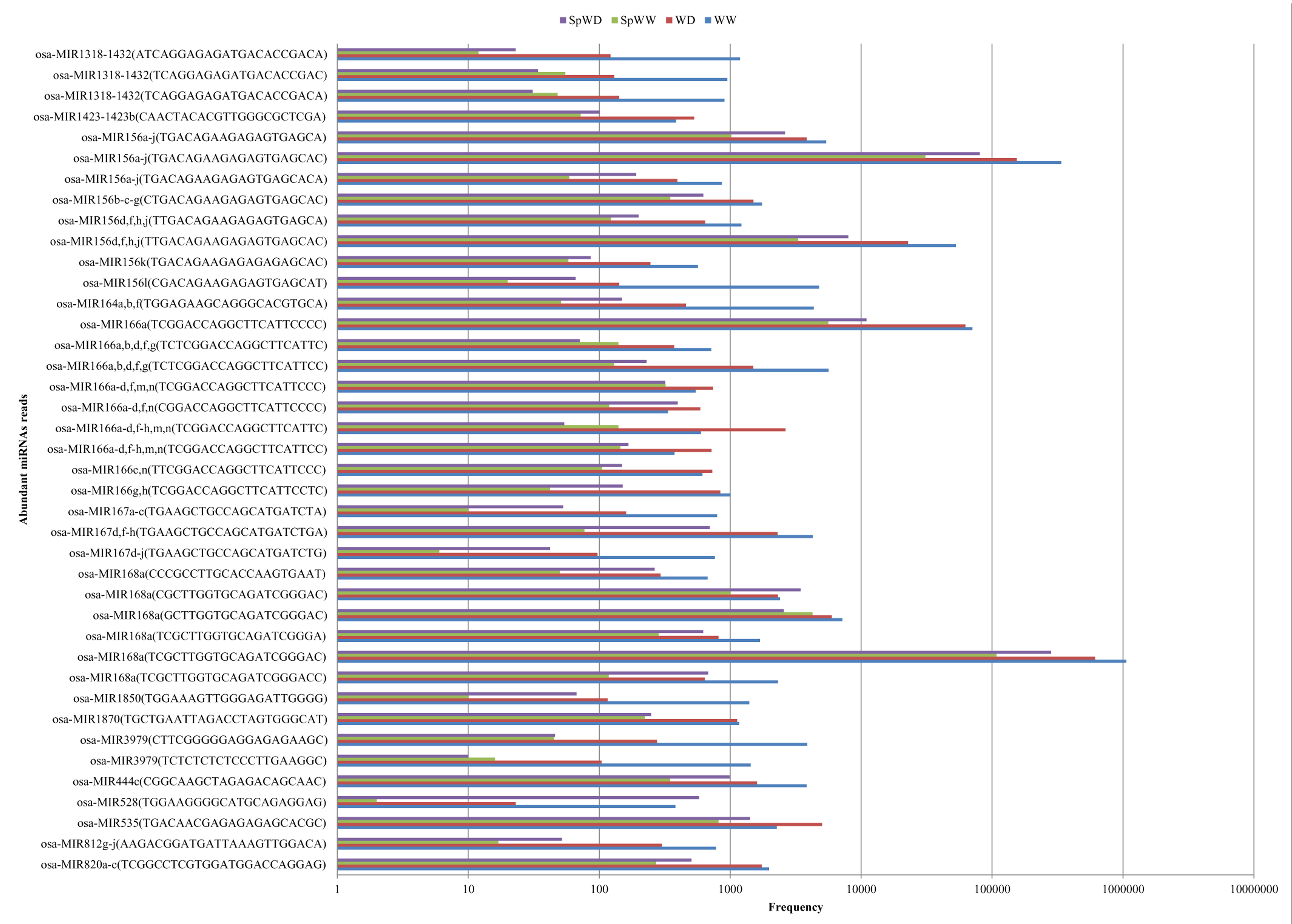

Supplement: S3 Fig — Y-axis indicates abundant miRNA members and X-axis indicates counts of miRNAs that are changed to logarithmic scale with base 10. Figure indicates that miR168, miR156 and miR166 are most abundant miRNAs. (TIF) [file pone.0156814.s003.tif]

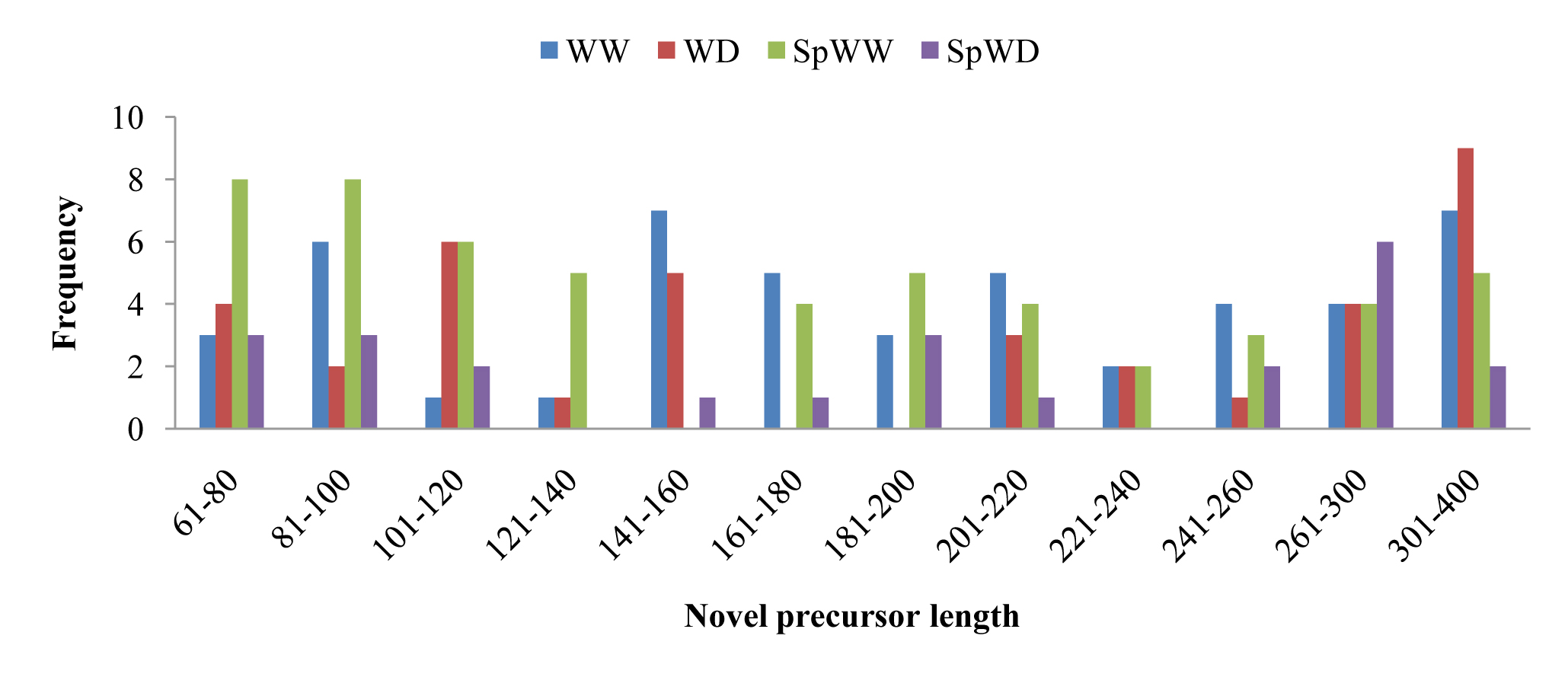

Supplement: S4 Fig — (TIF) [file pone.0156814.s004.tif]

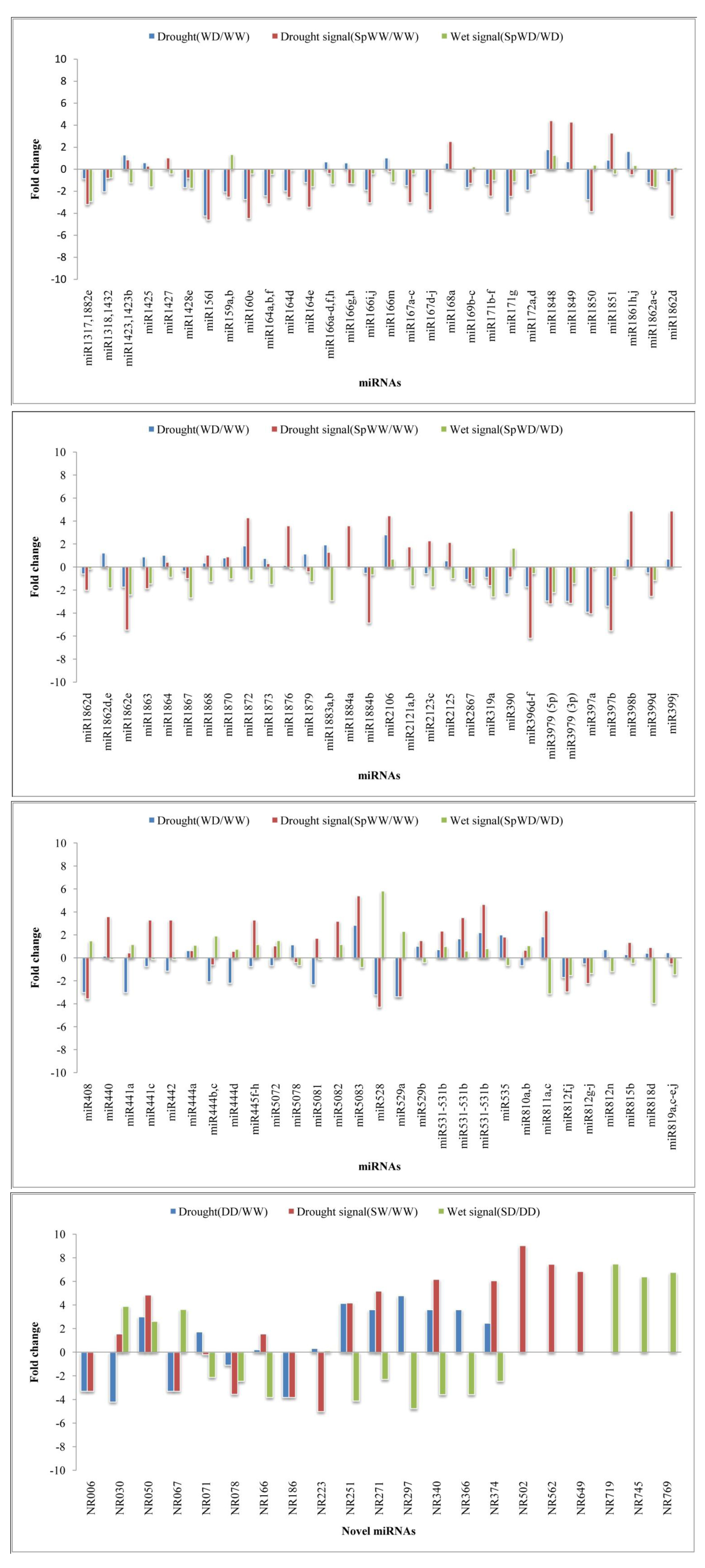

Supplement: S5 Fig — (TIF) [file pone.0156814.s005.tif]
